# The New Kilogram Definition and its Implications for High-Precision Mass Tolerance Classes

**DOI:** 10.6028/jres.118.016

**Published:** 2013-08-26

**Authors:** Patrick J Abbott, Zeina J Kubarych

**Affiliations:** National Institute of Standards and Technology, Gaithersburg, MD 20899

**Keywords:** kilogram redefinition, mass dissemination, uncertainty, weight classes for legal metrology

## Abstract

The SI unit of mass, the kilogram, is the only remaining artifact definition in the seven fundamental units of the SI system. It will be redefined in terms of the Planck constant as soon as certain experimental conditions, based on recommendations of the Consultative Committee for Mass and Related Quantities (CCM) are met. To better reflect reality, the redefinition will likely be accompanied by an increase in the uncertainties that National Metrology Institutes (NMIs) pass on to customers via artifact dissemination, which could have an impact on the reference standards that are used by secondary calibration laboratories if certain weight tolerances are adopted for use. This paper will compare the legal metrology requirements for precision mass calibration laboratories after the kilogram is redefined with the current capabilities based on the international prototype kilogram (IPK) realization of the kilogram.

## 1. Introduction

In their paper from 2003, Källgren and Pendrill make the point that “industrial metrology needs support from both scientific metrology and legal metrology in order to improve the quality of products and provide competitive and sustainable production [1].” Without a proper legal metrology framework, there is a risk of misapplication of scientific capability in the form of the selection of inappropriate precision for the task at hand. For example, one would not use a wooden meter stick to calibrate a gauge block. Conversely, a legal metrology system that indicates tolerances that are beyond the ability (or practicability) of scientific metrology is useless. For instance, specifying a chronometer with microsecond resolution to time a 100 meter footrace is a completely unreasonable and unhelpful use of technology. Mass metrology has benefitted greatly from the cooperation of scientific mass metrology and legal metrology. This is seen in the production of international standards that ensure appropriate application of the technical aspects of mass measurement and analysis to create a robust mass dissemination system.

Technological advances have enabled the invention of mass balances whose precision is one part in 10^10^ at the 1 kg level, which is a factor of 70 better than the smallest uncertainty that is ascribed by the International Bureau of Weights and Measures (BIPM) to the calibration of 1 kg platinum-iridium national prototypes [2]. Similarly, the OIML R111 standard’s [3] tightest tolerance is ± 0.083 mg (*k* = 1) at the 1 kg level for Class E1 weights which is a factor of 800 larger than the precision of the best balances. From these examples, it is clear that weighing instruments have become much better than any legal metrology requirement would dictate for industrial applications. This state of affairs will be extremely important as redefinition of the unit of mass is considered in the coming years.

## 2. Weight Classes for Legal Metrology

Precision weights must follow strict legal metrological and technical specifications that are spelled out in documents such as OIML R 111-1 and ASTM E-617 [3,4]. These documents provide detailed specifications for the manufacture of weights and weight sets along with guidance for correct usage. Among the qualities and quantities specified are: material, shape, density, surface finish, tolerance about the nominal value, uncertainty of the conventional mass, electrical and magnetic properties, and identifying markings. Currently, the most stringent specifications are placed on OIML Class E1 weights. According to OIML R 111-1, these weights are “intended to ensure traceability between national mass standards (with values derived from the International Prototype of the kilogram) and weights of class E2 and lower. Class E1 weights or weight sets shall be accompanied by a calibration certificate” [3]. In providing traceability to national mass standards, the permitted standard uncertainty of the conventional mass for E1 class weights must be tighter than that of class E2, yet not be so stringent as to make it difficult to calibrate with the national standards themselves. The permitted expanded uncertainties, *U* (*k* = 2), for the conventional mass of E1 and E2 weights are mandated to be less than or equal to one-third of the maximum permissible error on the nominal mass value. Table 1 shows these values for classes E1 and E2 at a nominal mass of 1 kg.

## 3. Mass Dissemination Chain

**Fig. 1:**
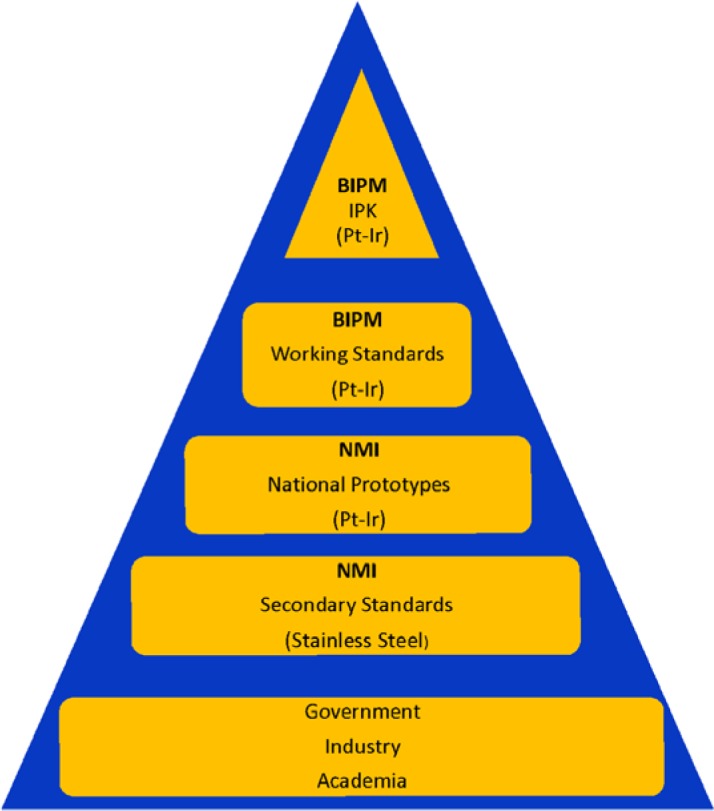
Diagram of mass dissemination from the IPK to end users.

The dissemination of the international system of units (SI) relies on a proper sequence of calibrations that begin at a given unit’s realization. In the case of mass, the definition of the unit and its realization are achieved by an artifact. This definition has served the measurement world since 1889 and is the only remaining artifact-based realization of an SI quantity. It reads as follows: “The kilogram is the unit of mass; it is equal to the mass of the international prototype of the kilogram [5].” The international prototype of the kilogram (IPK) is a platinum-iridium cylinder that is 39 mm in diameter by 39 mm in height. It resides in a vault at the BIPM and is rarely used. As such, the unit of mass can only be realized at the BIPM, and must be disseminated to the world through comparisons with BIPM working standards that are traceable to the IPK. A diagram of the current chain of dissemination is shown in Fig. 1.

By definition, the IPK has no uncertainty. Its mass is always exactly one kilogram after cleaning, even though its mass is known to be changing with time relative to other similar standards [6]. Therefore, the first component of uncertainty in the dissemination chain comes from the BIPM’s transfer of the mass unit to its own platinum-iridium working standards. Referring to Fig. 1, every vertical step downward adds uncertainty to the calibration result. Successful dissemination of the unit of mass requires that every vertical level in the dissemination pyramid shown in Fig. 1 receive an accurate calibration, traceable to the IPK, with a quantified uncertainty. The uncertainties associated with the current dissemination of the kilogram from the BIPM easily allow careful laboratories to disseminate multiples and submultiples of the kilogram at the E1 level. It will be seen in the next section that this is a vital requirement for any new kilogram definition.

## 4. Redefinition of the Kilogram

The international community has agreed to redefine the kilogram due to a long-term drift in the IPK relative to its official copies and in order to harmonize the kilogram definition with proposals for a “New SI” system of units based on fundamental constants [7]. The new definition will be in terms of the Planck constant, which will be fixed and have no uncertainty. An international effort is currently underway to measure the Planck constant using two different types of experiments: the watt balance [8–12] and the International Avogadro Coordination (IAC) project [13]. The IAC will actually determine the Avogadro constant using the x ray crystal density (XRCD) method, but from this the Planck constant may be calculated with negligible additional uncertainty. The proposed conditions for redefinition are as follows [14]:

1. At least three independent experimental results should yield values of the relevant constants with relative uncertainties not larger than 5 parts in 10^8^. One of these results should be derived from work being carried out by the International Avogadro Coordination (IAC) project. At least one of these results should have a relative standard uncertainty not larger than 2 parts in 10^8^.
2. Values of the Planck and Avogadro constants provided by these experiments should be consistent at the 95 % level of confidence.
3. Traceability of BIPM prototypes to the IPK should be confirmed.

These conditions have not yet been met, but they are very likely to be met within an acceptable level in the next few years given the number of watt balance experiments that are either running or near-running, along with improvements in the IAC project. The use of the word “should” in the above three conditions leaves room for deviations from the exact values stated. When redefinition occurs, the final approval of which will be the responsibility of the International Committee for Weights and Measures (CIPM) and the General Conference on Weights and Measures (CGPM), the magnitude of the kilogram will be defined by the exact numerical value assigned to the Planck constant when expressed in its SI unit J s. This value will have no uncertainty, but the uncertainty that was formerly ascribed to the Planck constant by the watt balance and IAC experiments before the redefinition will become the uncertainty of the IPK. Going forward, the uncertainties of realizations of the kilogram will depend on the uncertainties of current and future watt balance and XRCD experiments. The value of the Planck constant to be adopted to define the kilogram will be determined by CODATA [15] using all relevant experimental results.

## 5. Mass Dissemination after Redefinition

### 5.1 New Dissemination Uncertainty

The most important effect on the dissemination chain after redefinition will occur as a result of the nonzero uncertainty that will be placed on the best realization of the kilogram. Gläser et. al present data indicative of possible changes in uncertainty propagation after redefinition [16] in their Table 5. Two of these scenarios are summarized in our Table 2. It should be noted that reference [16] includes instability estimates of mass artifacts to better reflect realistic long-term behavior.

The requirements for class E1 calibration are met in the case where the starting uncertainty from BIPM 0.020 mg and are even met for the case of 0.050 mg starting uncertainty. This is not coincidental, as the criteria for redefinition presented by the CCM to the CGPM were made such that E1 uncertainties could still be met after redefinition.

### 5.2 Implications for a New Class of Legal Metrology

Some manufacturers of precision weights currently offer “Class E0” products, which are specified to have about one-third the maximum permissible error of Class E1 [17]. It is important to point out that as of 2012 this E0 class (or its equivalent) is not recognized by OIML R-111 or any other standards document. Table 3 compares the permissible errors and uncertainties for Class E1 and “Class E0” for a nominal mass of 1 kg. Comparing Tables 2 and 3, it is clear that even the case of a 0.020 mg uncertainty of the best realization of the kilogram after redefinition is too large to permit the dissemination of a “Class E0” tolerance down to laboratories that are currently accredited for E1 tolerances. Therefore, any claimed advantage in uncertainty that these weights may offer will not be able to be verified after redefinition [18]. This is a case where legal metrology and scientific metrology are not in harmony. The uncertainty of the new kilogram definition will be too large to stay within the prescribed tolerance for “Class E0”. Moving forward, the following questions need to be answered: “What is the industrial need for an “E0” class”; and, “Is the understanding of the factors that affect the stability of mass artifacts sufficient to actually produce weights having uncertainties that are one-third those of class E1?” Without a clearly identifiable need, the legal metrology is not necessary, and without quantitative evidence that such weights can be made, the legal metrology is not possible.

## 6. Summary and Discussion

Within the next few years, it is likely that the internationally agreed-upon conditions for redefining the kilogram in terms of Planck’s constant will be met to an acceptable level, and for the first time in the history of the SI, the primary realization of the kilogram will rightfully have an uncertainty ascribed to it. This uncertainty will be propagated along the mass dissemination chain and will likely result in larger calibration uncertainties than are currently assigned for customers of NMIs. The increase in uncertainty will not affect the ability to disseminate mass at the OIML Class E1 level, as the conditions for redefinition of the kilogram were carefully chosen for this reason, thereby preventing any “jump” or discontinuity in the mass scale. However, the situation is different for the “Class E0” weights that have appeared in the past few years. These have been given a very tight maximum permissible error that is in fact one-third of that of Class E1. Under the current mass dissemination system beginning with the IPK (and its zero uncertainty by definition), calibration uncertainties at NMIs are less than the tolerances of “Class E0” weights, so their calibration is possible. If realistic drift uncertainties were assigned to the IPK and other working standards, the small uncertainty calibrations that are required for “Class 0” weights would be much more tenuous, and the length of time required for an E0 weight to drift out of tolerance may be short. Under a new Planck constant based kilogram and assuming realistic estimates for artifact instabilities, calibration uncertainty estimates have been calculated that will clearly not permit the calibration of “Class E0” weights at the kilogram level. With time, the uncertainties associated with a Planck constant-based kilogram will improve, perhaps to the point where very small tolerance weight classes such as “E0” may be effectively calibrated. Even then, drift uncertainties may dictate that such weights be calibrated much more frequently than other mass classes.

## Figures and Tables

**Table 1:** Permissible errors and uncertainties for Class E1 and Class E2 for 1 kg

	Class E1 1 kg	Class E2 1 kg
Maximum permissible error	0.500 mg	1.600 mg
Maximum expanded uncertainty (*k* = 2)	0.167 mg	0.533 mg
Maximum standard uncertainty	0.083 mg	0.266 mg

**Table 2:** Uncertainty (*k* = 1) propagation from the best realization of the kilogram after redefinition, down to class E1 weights

Level in Dissemination Chain	Combined Standard Uncertainty (mg)	
	Case 1	Case 2
Best realization from Planck const.	0.020	0.050
BIPM Working Standards	0.030	0.071
NMI National Prototypes	0.030	0.071
NMI Secondary Standards	0.032	0.071
Reference Standards of E1Labs	0.044	0.077

**Table 3:** Comparison of permissible errors and uncertainties for Class E1 and “Class E0” for 1 kg nominal mas

	Class E1 1 kg	“Class E0” 1 kg

Maximum permissible error (mg)	0.500	0.150
Maximum expanded uncertainty (*k* = 2) (mg)	0.167	0.050
Maximum standard uncertainty (mg)	0.083	0.025
